# Pathological hypertrophy reverses *β*_2_-adrenergic receptor-induced angiogenesis in mouse heart

**DOI:** 10.14814/phy2.12340

**Published:** 2015-03-16

**Authors:** Qi Xu, Nicole L Jennings, Kenneth Sim, Lisa Chang, Xiao-Ming Gao, Helen Kiriazis, Ying Ying Lee, My-Nhan Nguyen, Elizabeth A Woodcock, You-Yi Zhang, Assam El-Osta, Anthony M Dart, Xiao-Jun Du

**Affiliations:** 1 Baker IDI Heart and Diabetes InstituteMelbourne, Victoria, Australia; 2 Institute of Cardiovascular Sciences, Peking University Health Science CenterBeijing, China; 3 Alfred Heart Centre, the Alfred HospitalMelbourne, Victoria, Australia; 4 Central Clinical School, Monash UniversityMelbourne, Victoria, Australia

**Keywords:** Angiogenesis, cAMP-responsive-element-binding protein, heart failure, hypertrophy, pressure overload, *β*_2_-adrenoceptor

## Abstract

*β*-adrenergic activation and angiogenesis are pivotal for myocardial function but the link between both events remains unclear. The aim of this study was to explore the cardiac angiogenesis profile in a mouse model with cardiomyocyte-restricted overexpression of *β*_2_-adrenoceptors (*β*_2_-TG), and the effect of cardiac pressure overload. *β*_2_-TG mice had heightened cardiac angiogenesis, which was essential for maintenance of the hypercontractile phenotype seen in this model. Relative to controls, cardiomyocytes of *β*_2_-TGs showed upregulated expression of vascular endothelial growth factor (VEGF), heightened phosphorylation of cAMP-responsive-element-binding protein (CREB), and increased recruitment of phospho-CREB, CREB-binding protein (CBP), and p300 to the VEGF promoter. However, when hearts were subjected to pressure overload by transverse aortic constriction (TAC), angiogenic signaling in *β*_2_-TGs was inhibited within 1 week after TAC. β_2_-TG hearts, but not controls, exposed to pressure overload for 1–2 weeks showed significant increases from baseline in phosphorylation of Ca^2+^/calmodulin-dependent kinase II (CaMKIIδ) and protein expression of p53, reduction in CREB phosphorylation, and reduced abundance of phospho-CREB, p300 and CBP recruited to the CREB-responsive element (CRE) site of VEGF promoter. These changes were associated with reduction in both VEGF expression and capillary density. While non-TG mice with TAC developed compensatory hypertrophy, (_2_-TGs exhibited exaggerated hypertrophic growth at week-1 post-TAC, followed by LV dilatation and reduced fractional shortening measured by serial echocardiography. In conclusion, angiogenesis was enhanced by the cardiomyocyte (_2_AR/CREB/VEGF signaling pathway. Pressure overload rapidly inhibited this signaling, likely as a consequence of activated CaMKII and p53, leading to impaired angiogenesis and functional decompensation.

## Introduction

Cardiac angiogenesis is vital for the preservation of myocardial function and the development of adaptive hypertrophy, and its impairment leads to heart failure (HF) (Sano et al. 2007; May et al. 2008; Taimeh et al. 2013). To meet the enhanced metabolic demand and to overcome an increased diffusion distance due to cardiomyocyte hypertrophy, a proportional increase in capillary density is required (Sano et al. 2007). In adult hearts, microvascular growth involves sprouting, intussusception, bridging, and intercalation of endothelial cells from existing vessels. Formation of new capillaries would preserve cardiac function and, conversely, the failure of capillary sprouting in settings of myocardial hypertrophy or heightened cardiac workload results in myocardial hypoxia and energy deficiency, eventually leading to dysfunction and HF (Heineke et al. 2007; Hilfiker-Kleiner et al. 2007).

Cardiomyocytes form a key source of vascular endothelial growth factor (VEGF) (Giordano et al. 2001; May et al. 2008; Taimeh et al. 2013). VEGF family of molecules, particularly VEGF-A, is critical for all aspects of angiogenesis and thought to be the primary angiogenic molecule mediating the crosstalk between cardiomyocytes and endothelial cells (Sano et al. 2007; Arany et al. 2008; May et al. 2008). Indeed, cardiomyocyte-specific knockout of VEGF-A resulted in reduced capillary density, ventricular thinning, and dysfunction, suggesting cardiomyocytes as a key source of VEGF-A (Giordano et al. 2001).

Activation of the sympatho-*β*-adrenergic system under stress conditions is important for the heart. Preclinical studies document that, unlike *β*_1_-adrenergic receptor (AR), use of *β*_2_AR agonists(Ahmet et al. 2008; Talan et al. 2011) or virally mediated *β*_2_AR overexpression (Akhter et al. 1997; Tomiyasu et al. 2000; Jones et al. 2004; Iaccarino et al. 2005) have therapeutic potential by promoting cardiac function in various disease settings. The salutary nature of *β*_2_-adrenergic signaling is indicated by Patterson et al. (2004) showing exacerbated cardiomyopathy and mortality in *β*_2_AR knockout than control animals receiving chronic treatment with isoproterenol. While a majority of studies using either *β*_2_-AR agonists or cardiac *β*_2_-AR gene transfection have shown beneficial effects (Tomiyasu et al. 2000; Patterson et al. 2004; Iaccarino et al. 2005; Ahmet et al. 2008; Chakir et al. 2011; Rengo et al. 2012), albeit has been no consensus on the therapeutic potential for heart disease through *β*_2_-AR activation (Talan et al. 2011; Zhu et al. 2012). Studies in cancer tissues have revealed that activation of *β*AR, particularly *β*_2_AR, promotes angiogenesis (Thaker et al. 2006; Wu et al. 2007; Annabi et al. 2009; Perez-Sayans et al. 2010; Zhang et al. 2010) and hence use of *β*_2_-antagonists are effective as anticancer therapy by suppressing VEGF expression (Yamaoka et al. 1993; Wu et al. 2007; Perez-Sayans et al. 2010; Zhang et al. 2010; Pasquier et al. 2013). Both *β*-adrenergic signaling and angiogenesis are critical for cardiac physiology and disease adaptation. However, interaction between both systems in the heart has received little attention.

The *β*_2_-AR TG model (TG4) was thoroughly studied during 1990s. In this model, a 200-fold increase in *β*_2_-AR density, driven by the *α*-myosin heavy chain promoter, results in spontaneous formation of the active confirmation of *β*_2_-AR (Bond et al. 1995), activating the downstream signaling pathway measured by increased levels of cAMP and activity of protein kinase A (PKA) (Milano et al. 1994; Bond et al. 1995). Whereas the inactive conformer is by far the predominant one, with a 200-fold increase in receptor density, the number of active *β*_2_-receptors would be high enough to support a full functional stimulation (i.e., increase in heart rate and cardiac contractility) (Milano et al. 1994; Du et al. 1996; Xu et al. 2011). Using a mouse model of cardiomyocyte-restricted overexpression of *β*_2_-AR, we addressed the hypothesis that cardiomyocyte *β*_2_AR signaling promotes angiogenesis by upregulation of VEGF in a paracrine fashion, which is critical for functional support and disease adaptation. We also explored whether such *β*_2_AR-mediated angiogenesis signaling operates under diseased conditions, that is, pressure-overload hypertrophy.

## Materials and Methods

### Animals, drug treatment, and surgery

The *β*_2_-TG and wild-type (WT) littermate mice and on a C57Bl6 × SJL mixed background were used in this study. Our previous studies showed age-dependent development of cardiomyopathy phenotype in the *β*_2_-TG mice starting from 8 months (Du et al. 2000b; Gao et al. 2003; Xu et al. 2011). We therefore used 3-month-old male mice free of cardiomyopathy. Mice were injected with an antiangiogenic agent TNP-470 (30 mg/kg, s.c. every second day) or vehicle for 2 weeks. TNP-470 is an analog of antibiotic fumagillin and able to inhibit endothelial cell proliferation (Yamaoka et al. 1993). Another batch of *β*_2_-TG and WT were anesthetized with ketamine/xylazine/atropine (100/20/1.2 mg/kg, respectively, i.p.), and subject to sham surgery or transverse aortic constriction (TAC) with the lumen size narrowed from 1 to 0.5 mm, as previously described (Xu et al. 2008). Experimental procedures were approved by a local Animal Ethics Committee and the investigation conforms to the Australian Code of Practice for the Care and Use of Animals for Scientific Purposes.

### Echocardiography

Anesthesia was maintained with 1.7% isoflurane. Echocardiography was performed using a Philips iE33 ultrasound machine and a 15 MHz linear-transducer. Short-axis 2-D image of the left ventricle (LV) at the level of the papillary muscles and 2-D guided M-mode images were acquired digitally. Images were blindly analyzed as described previously (Xu et al. 2011). LV dimensions at end-diastole and end-systole (LVDd, LVDs), and wall thickness (WT*h*) were measured. Fractional shortening (FS), LV mass index (LVMI), and h/r ratio were calculated.

### Micromanometry, electrocardiogram (ECG), and organ weight

LV function was assessed by using 1.4F microtipped pressure–volume (P/V) catheter (SPR839) and the ARIA system (Millar Instruments Inc., Houston, TX). Anesthesia was maintained with 1.7% isoflurane. Chest-lead ECG was recorded. P/V data were collected at steady state and during a transient occlusion of the inferior vena cava (Xu et al. 2011). Heart rate, LV systolic pressure (LVSP), LV end-diastolic pressure (LVEDP), end-diastolic volume (EDV), and end-systolic volume (ESV) were measured. Ejection fraction (EF), stroke volume (SV), d*P*/d*t*_max_, d*P*/d*t*_min_, relaxation time (*τ*), ventricular end-systolic elastance (Ees), preload adjusted maximal power, d*P*/d*t*-EDV relationship, preload recruitable stroke work relationship (Mw), and end-diastolic or end-systolic pressure–volume relationship (EDPVR, ESPVR) were calculated. At termination, mice were killed and the heart was isolated and immersed in saline on ice. Heart chambers were dissected and weighed separately.

### Gene expression

RNA was extracted from the LV tissue using TRIzol® reagent (Life Technologies, Carlsbad, CA). VEGF mRNA was determined by SYBR Green polymerase chain reaction (PCR) with an ABI PRISM 7700 Sequence Detection System and normalized to the level of GAPDH (Xu et al. 2011). The sequence of primers are as follows: AAACGAAAGCGCAAGAAATC (forward) and ATGCTTTCTCCGCTCTGAAC (reverse).

### Immunoblotting

Nuclear extracts were prepared from LV tissue, as described previously (Harikrishnan et al. 2005; Chang et al. 2011). Nuclear extracts (10 *μ*g protein) were separated on 4–15% SDS-PAGE. Membranes were incubated with antibodies against VEGF (Santa Cruz Biotechnology, Dallas, TX), cAMP-responsive-element-binding protein (CREB, 1:1,000), phospho-CREB (Cell Signaling Technology, Danvers, MA, 1:1,000), CREB-binding protein (CBP), hypoxia-inducible factor-1*α* (HIF-1*α*, Santa Cruz Biotechnology, 1:500), p300 (Abcam, Cambridge, UK, 1:500) or *α*-tubulin (Sigma-Aldrich, St Louis, MO, 1:20,000), and exposed using enhanced chemiluminescence reagent. Total CaMKII*δ*, the predominant isoform of CalMKII in the heart, and Thr286 phospho-CaMKII*δ* were determined in protein extract from LV tissues using antibodies (Santa Cruz Biotechnology). Band intensity was quantified using Quantity One (version 4.5.2; Bio-Rad, Hercules, CA).

### Histology and immunohistochemistry

Hearts were fixed in 4% paraformaldehyde in phosphate-buffered saline (PBS), paraffin-embedded and serially sectioned (5 *μ*m). The sections were incubated with citrate buffer (pH = 6.0) at 95–100°C to retrieve antigens. For immunohistochemical staining of VEGF, sections were incubated with antibodies against VEGF (Santa Cruz Biotechnology, 1:33) overnight at 4°C, amplified and stained with EnVision™ G|2 System/AP (Dako, Glostrup, Denmark), and then counterstained with hematoxylin. To show the cell source of VEGF, sections were further incubated with Biotinylated Wheat Germ Agglutinin (WGA, plasma membrane staining, Vector Laboratories, Burlingame, CA, 1:250) for 1 h at 37°C, and amplified and stained with the avidin–biotin complex (VEctastain ABC Elite, Vector Laboratories). For immunohistochemical staining of phospho-CREB, sections were incubated with antibodies against phospho-CREB (Cell Signaling Technology, 1:100) overnight at 4°C, washed in PBST, incubated with secondary antibody (1:2,000) for 1 h at 37°C, amplified and stained with the avidin–biotin complex, and counterstained with hematoxylin. Images were captured with Olympus BX50 microscope and analyzed as described previously (Xu et al. 2008). To assess angiogenesis and cardiomyocyte hypertrophy, sections were stained sequentially with Alexa Fluor 568 conjugated isolectin B4 (GS-IB4; Life Technologies, Carlsbad, CA, 1:20, overnight at 4°C) and FITC-labeled WGA (1:50, Vector Laboratories, 2 h at room temperature). Fluorescence images were captured with Olympus BX61 fluorescence microscope and analyzed blindly by using the Image-pro plus 6.0 System. Cardiomyocyte cross-sectional area and number of cardiomyocytes and microvessels were measured, and the ratio of capillaries to cardiomyocytes was calculated.

### Cell culture and pharmacological inhibition of Ca^2+^/calmodulin-dependent protein kinase II

The rat cardiomyoblast cell line (H9C2) was purchased from ATCC®. Cells in culture were treated for 1–24 h with isoproterenol (1 *μ*mol/L, Sigma-Aldrich) or vehicle. Selective *β*_1_- or *β*_2_-antagonist, atenolol, and ICI-118551 (both at 1 *μ*mol/L, Sigma-Aldrich) were tested. Expression of VEGF was measured by real-time RT-PCR with results normalized by the reference gene glyceraldehyde 3-phosphate dehydrogenase (GAPDH). To test signaling mechanisms for isoproterenol regulated VEGF expression, we tested effects of the PKA inhibitor KT5720 (0.3 *μ*mol/L), Ca^2+^/calmodulin-dependent protein kinase II (CaMKII) inhibitory peptide myristoylated-AIP (10 *μ*mol/L; Merck-Millipore, Darmstadt, Germany) or inhibitor KN93 (10 *μ*mol/L; Calbiochem, Merck-Millipore). To determine onset of isoproterenol-induced hypertrophic growth, cell size was determined using Image J (NIH, USA) and expression at mRNA level of atrial natriuretic peptide (ANP) was measured. Expression at protein level of p53 was determined by immunoblotting using antibody from Santa Cruz Biotechnology.

### Chromatin immunoprecipitation (ChIP)

ChIP assays using LV tissue were performed as described previously (Chang et al. 2011). Briefly, an equal amount of LV tissue (∽50 mg) was diced in ice cold PBS, fixed in 1% paraformaldehyde for 10 min, and incubated with 0.1 M glycine to quench the crosslinking. Then LV tissues were lysed in a lysis buffer containing 1% SDS, 10 mmol/L EDTA and 50 mmol/L Tris-HCl (pH = 8.0) and protease inhibitor cocktail (Cayman), and sonicated for 30 min to obtain chromatin fragments ranging from 300 to 500 bp in length. Soluble chromatin was diluted (1:10) in ChIP buffer containing 0.01% SDS, 1.1% Triton X-100, 1.2 mmol/L EDTA, 167 mmol/L NaCl and 16.7 mmol/L Tris-HCl (pH = 8.0) and incubated with antibodies (Santa Cruz Biotechnology) against Ser133 phospho-CREB (1:100), CBP (1:50) and p300 (1:50), together with protein G magnetic beads (Dynabeads, Life Technologies) overnight at 4°C. Nonspecific antibodies were used as negative controls. The beads were washed five times with low salt buffer, high salt buffer, lithium chloride buffer, TE buffer (pH = 8.0) and TE buffer containing 0.01% SDS, and then reversed cross-linked and eluted in ChIP elution buffer containing 5 mmol/L EDTA, 50 mmol/L NaCl, 1% SDS, proteinase K, and 20 mmol/L Tris-HCl (pH = 7.5). Analysis of chromatin immunoprecipitation DNA samples was performed by quantitative PCR of the putative CRE site of VEGF promoter region (forward: 5′-GCATGCATGTGTGTGTGTGT-3′; reverse: 5′-ATCTGTGCACCCCTTCAAAC-3′) (Lee et al. 2009) with an ABI PRISM 7700 Sequence Detection System. Data are normalized to the nonspecific DNA precipitation and expressed as percentage of the input samples.

### Statistical analysis

Results are expressed as mean ± SEM, unless otherwise indicated. Between-group comparisons were made by one- or two-way ANOVA or two-way repeated measures ANOVA with a Bonferroni post hoc analysis using GraphPad Prism 5 (GraphPad Inc., San Diego, CA), as appropriate. *P *<* *0.05 was considered as statistically significant. Fisher's exact test was used to compare frequency of events.

## Results

### β_2_AR-Promoted VEGF expression in cardiomyocytes and angiogenesis *in vivo*

**Figure 1:**
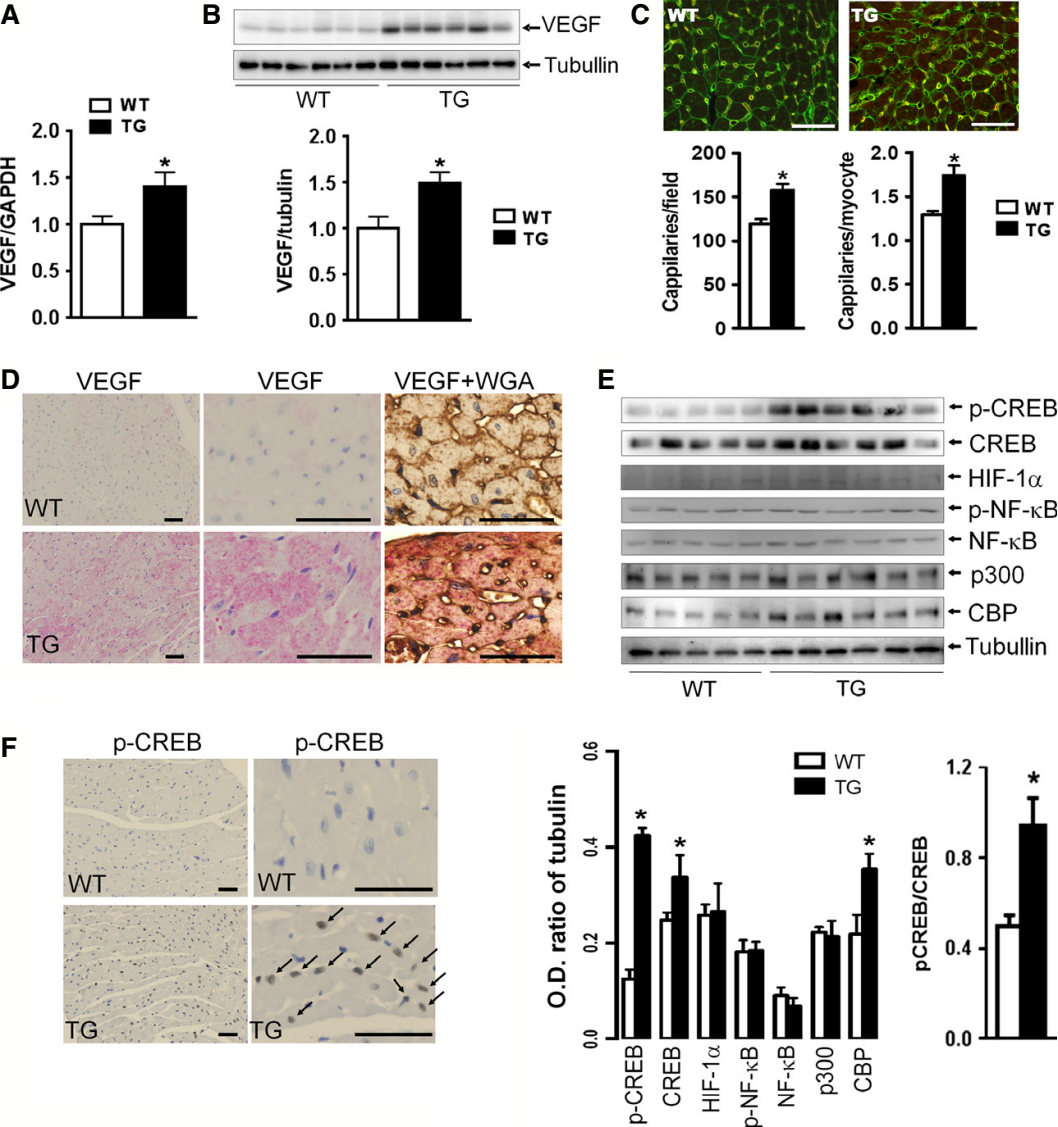
Enhanced VEGF expression and CREB activation in β_2_-TG left ventricular (LV) tissues. VEGF expression was significantly increased at mRNA (A) and protein levels (B) in TG hearts (*n* = 6–9/group). (C) representative immunohistochemical staining of capillaries of WT and *β*_2_-TG LVs. Bar = 50 *μ*m, *n* = 6 per group). (D) Representative immunohistochemical staining of VEGF protein (brown) or dual-stain of VEGF protein (pink) and cardiomyocytes (brown) in the LVs (*n* = 4 per group). WGA: wheat germ agglutinin. (E) Western blot analysis of p-CREB, CREB, HIF-1*α*, p-NF-*κ*B, NF-*κ*B, p300, CBP and tubulin in nuclear extracts of LVs of WT and *β*_2_-TG mice. *P <* *0.05 versus respective WT value. (F) Representative images of immunohistochemical staining of phospho-CREB (p-CREB) and nuclear accumulation of p-CREB (brown color indicated by arrows) in β_2_-TG cardiomyocytes (*n* = 3/group) with DAPI counterstain for nuclei. Bar = 50 *μ*m.

We initially studied the possibility of enhanced angiogenesis caused by transgenic activation of *β*_2_AR in cardiomyocytes. Compared with WT counterparts, VEGF expression in *β*_2_-TG hearts was 30% higher at mRNA and about 50% higher at the protein level (Fig.1A and B). Capillary density was 25% greater in the heart of *β*_2_-TG than WT counterparts (Fig.1C). Immunohistochemistry documented cardiomyocyte localization of enhanced VEGF expression (Fig.1D).

### CREB activation in *β*_2_-TG cardiomyocytes

CREB is a downstream molecule of the *β*_2_AR signaling pathway and is implicated in the regulation of VEGF expression (Lee et al. 2009). Immunoblotting analysis demonstrated a 3.3-fold increase in Ser^133^ phosphorylation of CREB in the myocardium of *β*_2_-TG mice (Fig.1E). Furthermore, immunohistochemical staining showed that phospho-CREB accumulated in cardiomyocyte nucleus of *β*_2_-TG than WT hearts (Fig.1F). CBP and p300 are coactivators of phospho-CREB. The expression levels of CBP, but not p300, was 60% higher in the LV of *β*_2_-TG than WT mice (Fig.1E). We also examined the expression of HIF-1*α* and nuclear localized NF-*κ*B, two important molecules regulating VEGF transcription. No differences were detected in the expression levels of both molecules in the LV of *β*_2_-TG and WT mice (Fig.1E).

### Increased recruitment of CREB, CBP, and p300 at the VEGF promoter in *β*_2_-TG hearts

**Figure 2:**
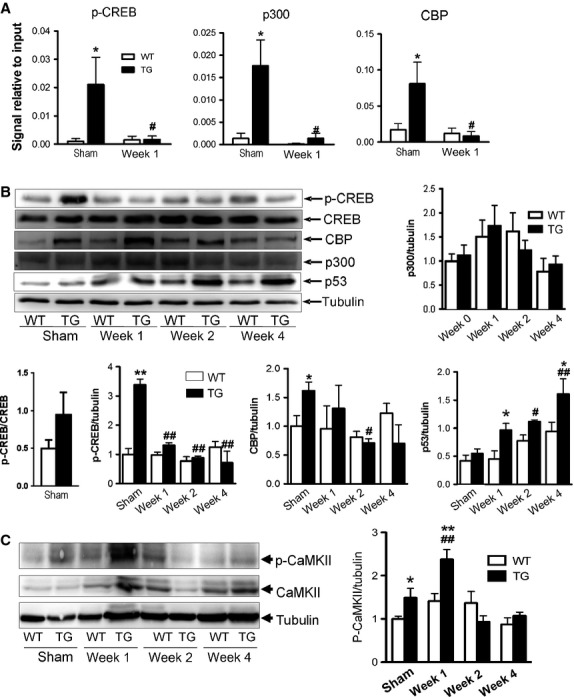
Identification of transcriptional factors associated with the VEGF promoter. (A) ChIP assays of p-CREB, CBP and p300 recruited to the VEGF promoter (*n* = 3 per group). (B) Representative immunoblotting of p-CREB, CREB, CBP, p300 and p53 in WT and *β*_2_-TG LVs at 1–4 weeks of transverse aortic constriction (TAC) or with sham operation. Bar graphs show densitometric analysis of protein expression. (C) Immunoblotting of total and Thr286 phospho-CaMKII*δ* in the LVs of WT and *β*_2_-TG mice (*n* = 5–6 per group) with sham surgery and at 1-4 weeks after TAC. Data were presented as changes relative to sham-operated WT mice (*n* = 3/group). *P <* *0.05, **P <* *0.01 versus respective WT group; ^#^*P *<* *0.05, ^##^*P *<* *0.01 versus sham operated *β*_2_-TG mice.

CREB is known to constitutively bind to the CRE site of the VEGF promoter (5′–TGAGGTGG–3′ between −1032 and −1025), and its transcriptional activity is enhanced by phosphorylation of CREB at Ser^133^, which allows for the recruitment of CBP and p300 to the CRE site. We next evaluated whether *β*_2_-TG hearts had an increased binding of phospho-CREB to the putative CRE site on the VEGF-A promoter (Accession No. U41383). We performed chromatin ChIP using antiphospho-CREB (Ser^133^) antibody with extracts of LVs from WT and *β*_2_-TG mice. There was a 3.5-fold increase in phospho-CREB enrichment with the CRE site of the VEGF promoter in LV extracts from *β*_2_-TG mice relative to that of WT mice. These studies also revealed that recruitment of CBP and p300 to the same promoter region was higher in *β*_2_-TG versus WT hearts (Fig.2A and B). Therefore, the transgenic activation of *β*_2_AR upregulates VEGF expression through phosphorylation of CREB with increased recruitment of coactivators, CBP and p300, to the VEGF promoter.

### Functional significance of enhanced angiogenesis in *β*_2_AR TG mice

**Figure 3:**
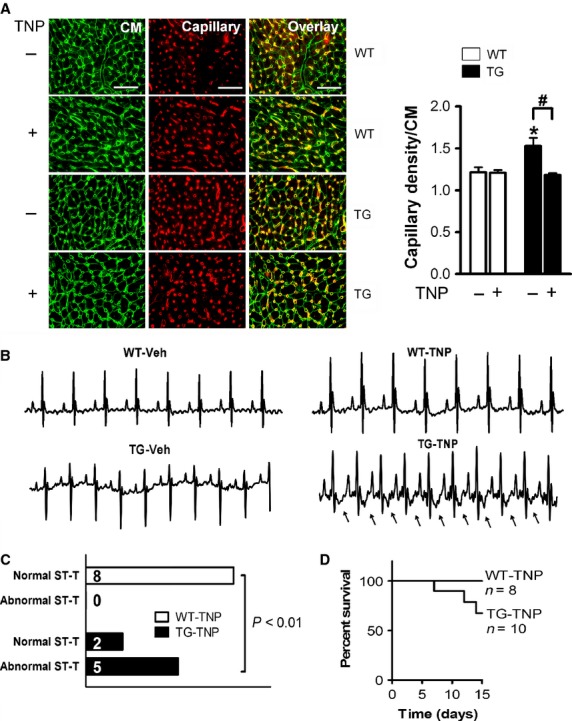
Treatment with the antiangiogenesis drug NP-470 suppressed cardiac angiogenesis in *β*_2_-TG hearts. WT and *β*_2_-TG mice were treated with vehicle or TNP-470 for 2 weeks. (A) representative images showing dual-stained cardiomyocytes (WGA, green) and endothelial cells (IB4, red) of the LVs (*n* = 4–7/group). Bar = 50 *μ*m. Capillary density was presented as the number of microvessels per cardiomyocyte. *P <* *0.05 versus WT controls; ^#^*P *<* *0.05. (B) Representative ECG traces after 2 weeks administration of TNP-470. Arrows indicate the typical ischemia-like ST-depression and T-wave inversion in the *β*_2_-TG mice treated with TNP-470. (C) Group analysis of ECG findings in WT and *β*_2_-TG mice (*n* = 7–8/group). (D) survival curve of WT and *β*_2_-TG mice during the 2 weeks period of TNP-470 treatment.

To explore the functional role of *β*_2_AR-induced angiogenesis, we tested the effect of TNP-470 as an inhibitor of angiogenesis (Yamaoka et al. 1993; Sano et al. 2007). TNP-470 or vehicle was given for a period of 2 weeks, and cardiac angiogenesis and function were examined subsequently. In WT mice, treatment with TNP-470 did not alter either cardiac functional parameters (Table1). TNP-470 treatment in *β*_2_-TG mice, however, reduced capillary density in the LVs to the level observed in WT controls (Fig.3A). Furthermore, administration of TNP-470 to *β*_2_-TG mice led to ECG changes reflecting myocardial ischemia (Fig.3B and C) and lower EF, increased EDV (Table3) and premature death (Fig.3D). All vehicle-treated WT (*n* = 9) and *β*_2_-TG mice (*n* = 8), as well as TNP-treated WT mice (*n* = 8), survived to the end of 2-week treatment period (Fig.3D). In 10 *β*_2_-TG mice treated with TNP-470, 3 mice died prematurely with signs of pulmonary congestion at autopsy (Fig.3D), and five of the seven surviving mice showed obvious ST depression and/or T-wave inversion in ECG (Fig.3C), changes not observed in WT mice receiving TNP-470 (*P* < 0.01 vs. TNP-470-treated TG mice).

**Table 1:** Body weight and normalized organ weights of WT and *β*_2_-TG mice at week 0–4 after transverse aortic constriction (TAC)

	WT				*β*_2_-TG			
	Week 0	Week 1	Week 2	Week 4	Week 0	Week 1	Week 2	Week 4
Number	10	12	8	12	8	7	12	14
Body weight, g	31 ± 1	28 ± 1	29 ± 1	30 ± 1	30 ± 2	28 ± 1	30 ± 1	30 ± 1
LV/BW, mg/g	3.2 ± 0.2	3.5 ± 0.1	3.8 ± 0.1#	4.3 ± 0.1#	3.0 ± 0.2	3.9 ± 0.1*#	4.4 ± 0.2*#	4.4 ± 0.1#
RV/BW, mg/g	0.72 ± 0.04	0.76 ± 0.02	0.65 ± 0.04	0.69 ± 0.03	0.70 ± 0.03	0.76 ± 0.03	0.70 ± 0.04	0.73 ± 0.02
Atria/BW, mg/g	0.26 ± 0.02	0.31 ± 0.02	0.29 ± 0.02	0.28 ± 0.02	0.26 ± 0.02	0.32 ± 0.02	0.40 ± 0.05	0.42 ± 0.03*#
Lung weight/BW, mg/g	5.1 ± 0.1	5.6 ± 0.2	5.0 ± 0.1	5.1 ± 0.2	4.9 ± 0.3	5.5 ± 0.3	6.0 ± 0.5	6.2 ± 0.3*#

*P < 0.05 versus respective WT groups; ^#^*P* < 0.05 versus WT or *β*_2_-TG mice at week-0, respectively.

**Figure 4:**
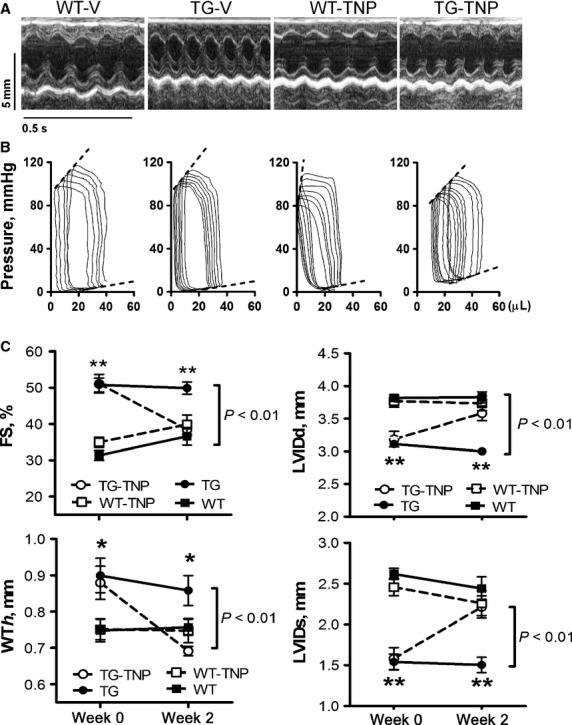
Adverse functional consequence of TNP-470 treatment only in *β*_2_-TG mice. Representative M-mode echocardiographic tracings from short-axis LV 2D images (A) and pressure–volume loops (B) from WT and *β*_2_-TG mice after 2 weeks treatment with vehicle or TNP-470. (C) Echocardiographic analysis of mice before and after treatment with vehicle or TNP-470 (*n* = 8–9/group). FS, fractional shortening; LVDd and LVDs, LV dimensions at end-diastole and end-systole, respectively; WT*h*, ventricular wall thickness at end-diastole. *P <* *0.05, **P <* *0.01 respective TG versus WT group. *P* values indicate within-group comparison between WK0 and WK2 in TG-TNP group.

Functionally, relative to that of WT counterparts, vehicle-treated TG mice showed elevated levels of contractile function, active relaxation at diastole, and wall thickness (WT*h*), while reduced EDV, ESV, LVDd, and LVDs (Fig.4, Table1). Administration of TNP-470 for 2 weeks led to overt LV dysfunction and dilatation, evidenced by significant reduction in FS, WT*h*, EF, Ees, d*P*/d*t*-EDV relationship, preload-adjusted maximal power, and M_w_, together with a markedly increased LV dimension or volumes at systole and diastole (all *P *<* *0.05) in comparison either with vehicle-treated TG mice or TNP-treated WT mice, as measured by echocardiography or pressure–volume loops (Fig.4, Table1).

### Pressure overload exacerbated cardiac hypertrophy and dysfunction in *β*_2_-TG hearts

**Figure 5:**
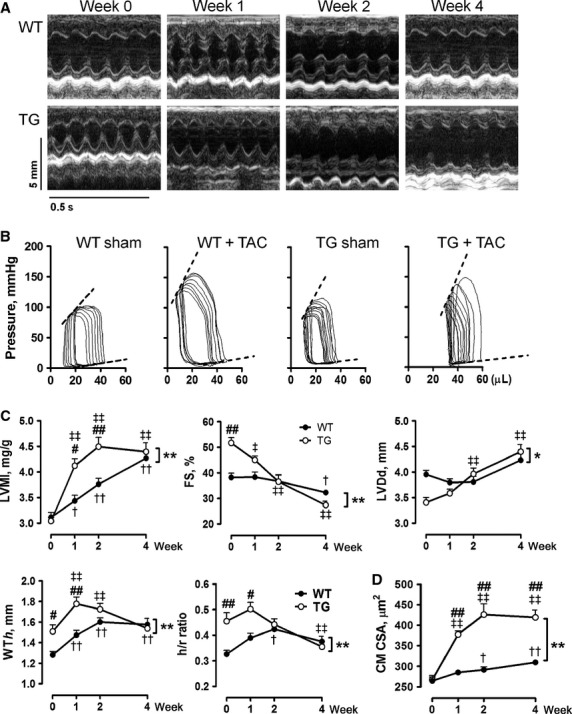
Cardiac hypertrophy and function after transverse aortic constriction (TAC) in wild-type (WT) and *β*_2_-TG mice. (A) Representative M-mode echocardiographic tracings from short-axis LV 2-D images from mice during 0–4 weeks of TAC and (B) representative pressure–volume loops of mice at week-4 post-TAC. Dotted lines indicate end-systolic or end-diastolic pressure/volume relationship. (C) Serial echocardiography on mice before and during 4 weeks of TAC (*n* = 7–13/group). LVMI, LV mass index; FS, fractional shortening; LVDd, LV dimensions at end-diastole; WT*h*, ventricular wall thickness at end-diastole; h/r ratio, ratio between WT*h* and LVDd. (D) cardiomyocyte cross-sectional area (CM CSA) in mice during 4 weeks of TAC (*n* = 5–7/group). *P <* *0.05, **P <* *0.01 between WT and *β*_2_-TG mice during 4 weeks of TAC; ^†^*P *<* *0.05, ^††^*P *<* *0.01 versus WT mice at week-0; ^‡^*P *<* *0.05, ^‡‡^*P *<* *0.01 versus TG mice at week-0; ^#^*P *<* *0.05, ^##^*P *<* *0.01 versus WT mice at respective time-point.

We examined cardiac function and hypertrophy at 1, 2, and 4 weeks after TAC (Fig.5). In WT mice, LV hypertrophy developed gradually, and FS was largely preserved during the 4-week period. Wall thickness, LVMI, and h/r ratio rose steadily post-TAC reaching a peak at week-2, and then declined slightly at week-4. There was a marginal LV dilatation (Fig.5A–C). In contrast, development of LV hypertrophy during the first 2 weeks after TAC was much faster in TG than WT mice, and leveled off thereafter (Fig.5A and C, Table2). FS decreased progressively, together with a progressive LV dilatation from 1-week post-TAC. Wall thickness and h/r ratio were unchanged at week-1 post-TAC and steadily fell thereafter (Fig.5A and C).

**Table 2:** Hemodynamic parameters of WT and *β*_2_-TG mice at 4 weeks after sham operation or TAC

	WT		TG	
	Sham	TAC	Sham	TAC
Number	6	5	5	7
Heart rate, beats/min	480 ± 25	478 ± 25	583 ± 15*	556 ± 13*
LV systolic pressure, mmHg	110 ± 6	153 ± 8#	94 ± 1	145 ± 14#
End-diastolic pressure, mmHg	9 ± 1	10 ± 2	10 ± 2	14 ± 3
End-diastolic volume, *μ*L	42 ± 2	42 ± 2	36 ± 1*	56 ± 6#
End-systolic volume, *μ*L	22 ± 2	21 ± 2	13 ± 1*	43 ± 8*#
Ejection fraction, %	47 ± 3	49 ± 1	64 ± 3*	27 ± 8#
Stroke volume, *μ*L	20 ± 1	20 ± 1	23 ± 1	13 ± 3
d*P*/d*t*_max_, mmHg/S	9535 ± 753	10948 ± 804	11852 ± 768	10027 ± 938
d*P*/d*t*_min_, mmHg/s	8292 ± 461	10550 ± 664#	7638 ± 289	8808 ± 880
τ, ms	5.1 ± 0.1	5.4 ± 0.3	4.5 ± 0.1*	5.4 ± 0.3#
Ees, mmHg/*μ*L	3.0 ± 0.4	6.3 ± 0.7#	5.6 ± 0.9*	6.1 ± 1.3
Preload adjusted maximal power, mW/*μ*L	69 ± 7	88 ± 6	121 ± 11*	52 ± 12*#
d*P*/d*t*-EDV, mmHg/s/*μ*L	129 ± 15	206 ± 28#	346 ± 53*	211 ± 33#
Mw, erg/cm^3^ per 10^3^	63 ± 2	115 ± 21#	88 ± 6*	61 ± 7*#
EDPVR, mmHg/*μ*L	0.29 ± 0.05	0.28 ± 0.11	0.31 ± 0.10	0.59 ± 0.17

*P <* *0.05 versus WT mice. ^#^*P* < 0.05 versus sham-operated *β*_2_-TG mice. LV, left ventricle; Ees, ventricular end-systolic elastance; EDV, end-diastolic volume; Mw, preload recruitable stroke work relationship; EDPVR, end-diastolic pressure–volume relationship.

Compared with WT counterparts, *β*_2_-TG mice pre-TAC showed elevated heart rate, systolic function, and active relaxation at the diastole (Fig.5A–C, Table3). EDV, ESV, *τ* were lower, whereas EF, Ees, d*P*/d*t*-EDV relationship, preload-adjusted maximal power and M_w_ were higher in TG than WT mice. TAC resulted in similar degree of pressure overload to both WT and TG mice (Fig.5B, Table3). As shown in Table3, following 4 weeks pressure overload, WT mice showed noticeable increment in ESPVR, d*P*/d*t*-EDV relationship, and M_w_, whereas TG mice developed pronounced LV dysfunction and dilatation, evidenced by significant reduction in EF, d*P*/d*t*-EDV relationship, preload-adjusted maximal power, and M_w_, together with marked increase in EDV, ESV, and *τ* (all *P *<* *0.05), relative to values of TG mice pre-TAC or WT mice with TAC.

**Table 3:** Hemodynamic parameters of WT and *β*_2_-TG mice at 2 weeks after treatment with vehicle or TNP-470

	WT		TG	
	Vehicle	TNP470	Vehicle	TNP470
Number	5	7	5	6
Heart rate, beats/min	487 ± 1	474 ± 14	565 ± 9*	532 ± 23*
LV systolic pressure, mmHg	98 ± 6	96 ± 2	92 ± 7	93 ± 2
End-diastolic pressure, mmHg	6 ± 1	5 ± 0	6 ± 2	11 ± 1*
End-diastolic volume, *μ*L	38 ± 3	34 ± 3	33 ± 4	51 ± 2*#
End-systolic volume, *μ*L	19 ± 3	13 ± 3	10 ± 3	21 ± 2*#
Ejection Fraction, %	54 ± 5	66 ± 5	72 ± 4*	58 ± 3#
Stroke Volume, *μ*L	19 ± 1	21 ± 2	23 ± 2	30 ± 2*
d*P*/d*t*_max_, mmHg/s	9645 ± 314	10526 ± 360	10866 ± 667	10057 ± 784
d*P*/d*t*_min_, mmHg/s	7006 ± 415	7783 ± 291	8095 ± 673	7183 ± 662
τ, ms	6.7 ± 0.2	5.9 ± 0.3	5.2 ± 0.2*	6.9 ± 1.0
Ees, mmHg/*μ*L	2.0 ± 0.2	2.0 ± 0.3	4.3 ± 0.5*	2.1 ± 0.5#
Preload adjusted maximal power, mW/mL^2^	80 ± 13	83 ± 12	148 ± 36	48 ± 5*#
d*P*/d*t*-EDV, mmHg/s/*μ*L	152 ± 25	154 ± 16	397 ± 58*	95 ± 8*#
Mw, erg/cm^3^ per 10^3^	47 ± 5	62 ± 6	85 ± 12*	53 ± 6#
EDPVR, mmHg/*μ*L	0.28 ± 0.05	0.29 ± 0.06	0.37 ± 0.06	0.24 ± 0.08

*P < 0.05 versus WT mice; ^#^*P* < 0.05 versus vehicle-treated *β*_2_-TG mice. LV, left ventricle; Ees, ventricular end-systolic elastance; EDV. end-diastolic volume; Mw, preload recruitable stroke work relationship; EDPVR, end-diastolic pressure–volume relationship.

### Pressure overload abolished the augmented angiogenesis present in the β_2_-TG heart

**Figure 6:**
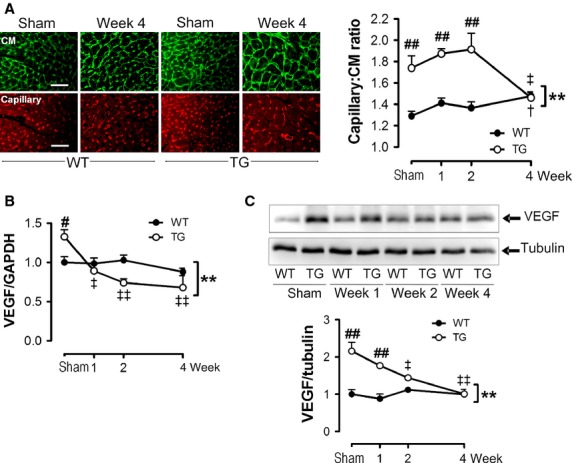
Abrogation of angiogenesis signaling in *β*_2_-TG hearts following pressure overload. (A) representative images of dual-staining with WGA for cell membrane (green) and IB4 for endothelial cells (red) of LVs of mice with sham-operation or TAC for 1, 2, and 4 weeks post-TAC (*n* = 5–7/group). Bar = 50 *μ*m. Line graphs show changes in capillary density as the ratio of number of microvessels per cardiomyocyte. (B) Representative Western blot of VEGF expression in WT and *β*_2_-TG LVs at 0–4 weeks of TAC with data quantified by densitometry, normalized by the level of tubulin and presented as changes relative to WT mice at week-0 (*n* = 3/group). C, Expression VEGF at mRNA level in the LVs of WT and *β*_2_-TG mice (*n* = 6–11 per group) before and during 4 weeks of TAC. **P <* *0.01 between WT and *β*_2_-TG mice during 4 weeks of TAC; ^†^*P *<* *0.05 versus WT mice at week-0; ^‡^*P *<* *0.05, ^‡‡^*P *<* *0.01 versus TG mice at week-0; ^#^*P *<* *0.05, ^##^*P *<* *0.01 versus WT mice at respective time-point.

We next studied the pattern of cardiac angiogenesis before and after TAC in WT and TG hearts. Capillary density was noticeably higher in the hearts from sham-operated *β*_2_-TG than WT mice (Fig.6A). VEGF expression at mRNA (Fig.6B) and protein levels (Fig.6C) was also higher in hearts of *β*_2_-TG than WT mice. Furthermore, in parallel with the strikingly hypertrophic growth and functional changes in TG mice after TAC, both capillary density per cardiomyocyte and VEGF protein were substantially lower than observed in sham-operated *β*_2_-TG mice. These levels were equivalent to or even lower than those of WT mice after 1–2 weeks (Fig.6A–C), albeit the VEGF mRNA declined progressively from 1-week post-TAC (Fig.6B and C). In WT hearts, VEGF expression was unchanged following TAC but capillary density was significantly increased at week-4 post-TAC versus sham group (Fig.6A).

### Pressure overload was associated with reduction in phospho-CREB, release of p300 and CBP from VEGF promoter, and activation of CaMKII

In keeping with the progressive decline in VEGF mRNA level in the LV of *β*_2_-TG mice after TAC, phosphorylation of CREB and the abundance of CBP were concurrently reduced (Fig.2A and B). Moreover, there was a dramatic dissociation of phospho-CREB, p300 and CBP from the CRE site of the VEGF promoter at week-1 post-TAC (Fig.2A). No changes were detected in the expression level of p300 throughout the 4-week period of TAC in either *β*_2_-TG or WT mice (Fig.2B). Interestingly, expression of p53 increased steadily in *β*_2_-TG hearts following TAC, becoming significant after week-2 (Fig.2B). Levels of total and p-CaMKII in LVs of *β*_2_-TG or WT mice with TAC were determined by immunoblotting. In contrast to unaltered levels of both parameters in WT hearts, marked elevation of p-CaMKII was observed in *β*_2_-TG hearts at week-1 post-TAC, but not in other time-points studied (Fig.2C).

### Biphasic regulation of VEGF expression by *β*-AR agonist and mechanisms in H9C2 cells

**Figure 7:**
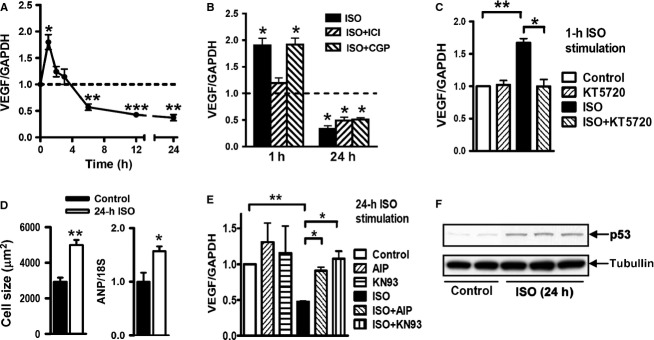
Biphasic regulation of the *β*-adrenergic activation on VEGF expression in the rat cardiomyocyte cell line (H9C2). (A) the *β*AR agonist isoproterenol (ISO, 1 *μ*mol/L) induced prompt upregulation of VEGF expression. This action was rapidly lost and replaced by a sustained downregulation during 6 to 24 h period. (B) ISO-mediated upregulation of VEGF expression was inhibited by the *β*_2_-AR agonist ICI118551 (1 *μ*mol/L) but not affected by the *β*_1_-AR agonist CGP12177 (1 *μ*mol/L). Both specific antagonists showed no effect on suppressed VEGF expression by 24-h ISO stimulation. *n* = 3 measures/group of two repeated experiments. (C) The upregulation of VEGF expression by 1-h ISO stimulation was blocked by the PKA inhibitor KT5720 (0.3 *μ*mol/L). (D) the downregulation of VEGF expression by 24-h ISO stimulation was abolished by CaMKII inhibitors myristoylated-AIP or KN93 (both at 10 *μ*mol/L, C). (E) Cellular hypertrophy stimulated by 24-h ISO measured by cell size and expression of atrial natriuretic peptide (ANP). *n* = 6 per group (F) upregulation of p53 expression by 24-h ISO stimulation. *n* = 6 independent measures/group. *P <* *0.05, **P <* *0.01, and ***P <* *0.001 versus respective control.

H9C2 cardiomyoblasts were stimulated with isoproterenol and expression of VEGF at mRNA level was measured at 1, 2, 3, 6, 12, and 24 h. A biphasic change in VEGF expression was observed (Fig.7A). The early increase in VEGF expression was blocked by ICI-118551 or PKA inhibitor KT5720, but unaffected by the *β*_1_-antagonist atenolol, suggesting *β*_2_-AR/PKA-mediated upregulation (Fig.7B). Both ICI-118551 and atenolol showed no effect on suppressed VEGF expression at 24 h after isoproterenol stimulation (Fig.7B). Isoproterenol stimulation is known to induce cellular hypertrophy. Indeed, we observed enlarged cell size and upregulated hypertrophy marker gene ANP at 24 h after isoproterenol treatment (Fig.7D). We tested effect of two independent CaMKII inhibitors, AIP and KN93, on VEGF expression. While having no effect on VEGF expression at baseline, both inhibitors abolished isoproterenol-induced suppression of VEGF expression (Fig.7E). Cells treated with isoproterenol for 24 h had elevated expression of p53 by immunoblotting (Fig.7F), a finding in consistent with that of the hypertrophic heart from *β*_2_-TG mice.

## Discussion

In this study, we demonstrated that (1) transgenic activation of cardiomyocyte *β*_2_AR promotes VEGF expression and cardiac angiogenesis via a paracrine mechanism, which is essential to meet the metabolic needs of increased heart rate and contractility in *β*_2_-TG hearts; (2) *β*_2_AR-mediated angiogenesis promotes LV hypertrophy in the early stage of pressure overload. A switch from upregulated to impaired angiogenesis, however, occurs shortly after TAC resulting in cardiac decompensation and HF; and (3) *β*_2_AR activation promotes VEGF expression by phosphorylation of CREB and subsequent recruitment of p300 and CBP to the VEGF promoter. This signaling pathway, however, is inhibited following pressure-overload hypertrophy, likely mediated by enhanced signaling of CaMKII and p53, providing an explanation for the functional decompensation and adverse ventricular remodeling in *β*_2_-TG hearts after TAC.

We demonstrated that cardiomyocyte *β*_2_ARs promote VEGF expression and modulate cardiac angiogenesis, providing evidence for the significance of *β*_2_AR in harmonizing cardiac function via a paracrine mechanism. *β*_2_ARs have been known to regulate vascular function as well as activation of endothelial cell function and growth largely via release of nitric oxide (Iaccarino et al. 2002; Ciccarelli et al. 2011; Martini et al. 2011; Rengo et al. 2012). The current studies show that cardiomyocytes form the key reservoir for VEGF that regulates cardiac angiogenesis following *β*_2_AR activation. A paracrine regulation of angiogenesis by *β*_2_AR has previously been reported in tumor, (Thaker et al. 2006; Wu et al. 2007; Perez-Sayans et al. 2010; Zhang et al. 2010; Pasquier et al. 2013) or the retina, (Martini et al. 2011) where activation of *β*_2_AR mediates release of angiogenic factors XZC from nonendothelial cells and subsequent neovascularization through a paracrine mechanism in vivo (Fredriksson et al. 2000; Thaker et al. 2006; Martini et al. 2011). In these studies, *β*-adrenergic blockade effectively prevented the release of angiogenic factors and neoangiogenesis.

We showed that transgenic activation of cardiomyocyte *β*_2_AR increases expression of VEGF, a key angiogenic molecule in coordinating the crosstalk between cardiomyocytes and endothelial cells. (Sano et al. 2007; Taimeh et al. 2013) Cardiomyocyte-specific knockout of VEGF resulted in reduced capillary density, ventricular wall thinning, and contractile dysfunction, suggesting a pivotal role of enhanced angiogenesis in the heart (Giordano et al. 2001). VEGF expression is modulated by several transcriptional factors, including HIF-1*α*, NF-*κ*B, and CREB. HIF-1*α* is the key transcription factor involved in VEGF responses during hypoxia, but it degrades rapidly under normoxic conditions (Liu and Simon 2004). NF-*κ*B is known to induce VEGF expression in macrophages and endothelial cells (Kiriakidis et al. 2003; Ciccarelli et al. 2011). CREB can upregulate VEGF expression either by forming a transcription complex with HIF-1*α* at the HRE site in the promoter region, (Wu et al. 2007) or acting alone on the CRE site of VEGF promoter (Lee et al. 2009). We found in the *β*_2_-TG hearts that cAMP-CREB signaling is activated without detectable changes in HIF-1*α* or NF-*κ*B, indicating that the phospho-CREB binding to the CRE site is the most likely mechanism responsible for enhanced VEGF transcription in *β*_2_-TG hearts.

Angiogenesis is one of the key determinants in the development of HF (Taimeh et al. 2013). Under basal conditions, the higher heart rate and contractility driven by transgenic activation of *β*_2_AR is dependent on concomitantly enhanced angiogenesis, as *β*_2_-TG but not WT mice respond to the angiogenic inhibitor TNP-470 with signs of cardiac ischemia, onset of premature death, impaired contractility, and LV dilatation. In the setting of pressure overload, the ratio of capillary:cardiomyocyte in the *β*_2_-TG heart is increased within the first 2 weeks. This change is apparently compensatory by meeting a higher energy demand due to pressure overload and increased diffusion distance due to cardiomyocyte hypertrophy. Our finding of a facilitated hypertrophic growth in *β*_2_-TG hearts together with increased capillary density supports the view that cardiac angiogenesis is a key factor for the development of pressure-overload hypertrophy and is crucial in preserving cardiac function, (Heineke et al. 2007; Hilfiker-Kleiner et al. 2007; Sano et al. 2007) as loss of such angiogenetic support results in rapid functional decompensation as seen in *β*_2_-TG mice with TAC.

While *β*_2_-TG mice have enhanced cardiac contractility, we previously found, in the setting of severe pressure overload, that this hypercontractile phenotype failed to preserve function, and, on the contrary, was associated with worsening of HF (Du et al. 2000a; Sheridan et al. 2000). Here, we revealed that under conditions of a moderate degree of pressure overload, *β*_2_-TG hearts responded with an exacerbated hypertrophic growth in the first 1–2 weeks after TAC, which peaked at week-2 and leveled off afterward. Importantly, the *β*_2_-TG mice with TAC developed LV dilatation and systolic dysfunction from week-1 post-TAC. The changes in *β*_2_-TG hearts pre- and post-TAC suggest that the *β*_2_AR stimulated hypercontractility and hypertrophic growth after pressure overload are underpinned by angiogenesis, which, however, rapidly attenuated post-TAC, resulting in cardiac dysfunction and adverse remodeling.

Following TAC, reduced expression of VEGF at mRNA and protein levels become evident within the first week whereas significant reduction in the density of capillaries was observed at week-4. Such discrepancy in the time-course of both parameters is very likely due to a turnover rate of capillaries that makes changes in capillary density lag behind the suppressed VEGF expression, which can occur rather acutely. This has been indicated by studies from other groups (Sano et al. 2007; May et al. 2008).

For therapeutic reasons, it is important to identify factors responsible for the inactivation of the *β*_2_AR/VEGF signaling cascade under diseased conditions. Recent studies suggest a detrimental role of CaMKII in mediating the transition from compensatory hypertrophy to HF, (Ling et al. 2009; Swaminathan et al. 2012) and in upregulating p53 (Toko et al. 2010). In the pressure-overload hypertrophic heart of *β*_2_-TG mice, we observed a marked increase in phosho-CaMKII at week-1 post-TAC, whereas the increase in p53 expression was evident at week 2 and week 4. This finding may suggest a sequential signaling event leading to suppression of VEGF expression. According to the proposed “coactivator-poor” model, (Kasper et al. 2010; Altarejos and Montminy 2011) there is only one putative CRE palindrome in the VEGF promoter. Thus, recruitment of CBP and p300 to the CRE site as well as the phosphorylation of CREB are crucial to VEGF expression following *β*_2_AR-promoted CREB phosphorylation in cardiomyocytes. Pressure overload is associated with suppression of CREB phosphorylation at serine^133^. In addition, p53, a key factor mediating cell arrest and apoptosis, (Green and Kroemer 2009) increased significantly in *β*_2_-TG hearts following TAC. p53 is known to directly interact with the KIX domain of CBP, and CREB facilitates this interaction (Giebler et al. 2000). As the transcriptional activation of VEGF is highly coactivator-dependent, the presence of p53 could lead to the formation of p53/CREB/CBP/p300 complex, forcing the release of the CREB/CBP/p300 complex from the VEGF promoter. Our experiments on H9C2 cells also showed early upregulation by the addition of isoproterenol, a response that was blocked by the *β*_2_-antagonist ICI-118551, whereas *β*_1_-blockade showed no effect. This finding suggests *β*_2_AR-stimulated VEGF expression and is in keeping with the fact that H9C2 cells express predominantly *β*_2_AR (71%) (Dangel et al. 1996). The subsequent suppression by isoproterenol of VEGF expression was accompanied by the development of cellular hypertrophy and can be abolished by the use of CaMKII inhibitors. Meanwhile, increased expression of p53 by hypertrophic cells was evident, a finding in consistent with our in vivo data.

Therapeutic potential of *β*_2_-AR overexpression is currently unclear and there has been no consensus on whether overexpression/activation of *β*_2_-AR is an effective and safe therapy for heart disease. In our study, we have made two important findings: (1) under physiological conditions, enhanced *β*_2_-AR signaling promotes cardiac angiogenesis; and (2) inactivation of such *β*_2_-AR/VEGF signaling under conditions of pressure-overload hypertrophy. As we discussed, the latter finding would bear therapeutic implication, for example, simultaneous *β*_2_-AR activation and inhibition of p53 and CaMKII. The documented *β*_2_AR/VEGF mechanism has therapeutic potential for heart disease. However, as we observed in mice with TAC, therapies enhancing *β*_2_AR signaling need to be combined with inhibitors of CaMKII and/or p53 activity to ensure the maintenance of such action. CaMKII and p53 have been regarded as therapeutic targets for heart disease (Sano et al. 2007; Toko et al. 2010; Anderson et al. 2011; Swaminathan et al. 2012). Our findings indicate alternatively mechanism by which activation of both molecules is associated with suppressed angiogenesis signaling via *β*_2_AR activation.

Our study has some limitations. First, our study lacks direct evidence from in vivo experiments on the significance of CaMKII and p53 in mediating inhibition of *β*_2_AR signaling with enhanced angiogenesis. Although our findings from cultured cell model support this notion, further works are required to ascertain this in vivo. Second, we only studied *β*_2_AR TG mice subjected to pressure-overload challenge. Whether the *β*_2_AR/VEGF signaling undergoes similar change remains to be addressed in other diseased conditions.

In conclusion, our data provide evidence for a link between *β*_2_AR and cardiac angiogenesis. Under physiological conditions, *β*_2_AR activation in cardiomyocytes promotes angiogenesis to preserve cardiac function via upregulated expression of VEGF, which is tightly controlled by the CREB/CBP/p300 transcriptional complex. Under conditions of pressure-overload hypertrophy, *β*_2_AR activation and the downstream signaling mediating VEGF/angiogenesis is uncoupled. This causes impaired angiogenesis, development of adverse remodeling and ultimately HF. Further studies are warranted to investigate whether restoration of the *β*_2_AR/CREB/VEGF signaling would be beneficial in HF management.

## Conflict of Interest

None declared.

## References

[b1] Ahmet I, Krawczyk M, Zhu W, Woo AY, Morrell C, Poosala S (2008). Cardioprotective and survival benefits of long-term combined therapy with *β*_2_-adrenoreceptor (AR) agonist and *β*_1_-AR blocker in dilated cardiomyopathy postmyocardial infarction. J. Pharmacol. Exp. Ther.

[b2] Akhter SA, Skaer CA, Kypson AP, McDonald PH, Peppel KC, Glower DD (1997). Restoration of *β*-adrenergic signaling in failing cardiac ventricular myocytes via adenoviral-mediated gene transfer. Proc. Natl Acad. Sci. USA.

[b3] Altarejos JY, Montminy M (2011). CREB and the CRTC co-activators: sensors for hormonal and metabolic signals. Nat. Rev. Mol. Cell Biol.

[b4] Anderson ME, Brown JH, Bers DM (2011). CaMKII in myocardial hypertrophy and heart failure. J. Mol. Cell. Cardiol.

[b5] Annabi B, Lachambre MP, Plouffe K, Moumdjian R, Beliveau R (2009). Propranolol adrenergic blockade inhibits human brain endothelial cells tubulogenesis and matrix metalloproteinase-9 secretion. Pharmacol. Res.

[b6] Arany Z, Foo SY, Ma Y, Ruas JL, Bommi-Reddy A, Girnun G (2008). HIF-independent regulation of VEGF and angiogenesis by the transcriptional coactivator PGC-1*α*. Nature.

[b7] Bond RA, Leff P, Johnson TD, Milano CA, Rockman HA, McMinn TR (1995). Physiological effects of inverse agonists in transgenic mice with myocardial overexpression of the *β*_2_-adrenoceptor. Nature.

[b8] Chakir K, Depry C, Dimaano VL, Zhu WZ, Vanderheyden M, Bartunek J (2011). G*α*s-biased *β*_2_-adrenergic receptor signaling from restoring synchronous contraction in the failing heart. Sci. Transl. Med.

[b9] Chang L, Kiriazis H, Gao XM, Du XJ, El-Osta A (2011). Cardiac genes show contextual SWI/SNF interactions with distinguishable gene activities. Epigenetics.

[b10] Ciccarelli M, Sorriento D, Cipolletta E, Santulli G, Fusco A, Zhou RH (2011). Impaired neoangiogenesis in *β*-adrenoceptor gene-deficient mice: restoration by intravascular human *β*-adrenoceptor gene transfer and role of NF*κ*B and CREB transcription factors. Br. J. Pharmacol.

[b11] Dangel V, Giray J, Ratge D, Wisser H (1996). Regulation of *β*-adrenoceptor density and mRNA levels in the rat heart cell-line H9c2. Biochem. J.

[b12] Du XJ, Vincan E, Woodcock DM, Milano CA, Dart AM, Woodcock EA (1996). Response to cardiac sympathetic activation in transgenic mice overexpressing *β*_2_-adrenergic receptor. Am. J. Physiol.

[b13] Du XJ, Autelitano DJ, Dilley RJ, Wang B, Dart AM, Woodcock EA (2000a). *β*_2_-adrenergic receptor overexpression exacerbates development of heart failure after aortic stenosis. Circulation.

[b14] Du XJ, Gao XM, Wang B, Jennings GL, Woodcock EA, Dart AM (2000b). Age-dependent cardiomyopathy and heart failure phenotype in mice overexpressing *β*_2_-adrenergic receptors in the heart. Cardiovasc. Res.

[b15] Fredriksson JM, Lindquist JM, Bronnikov GE, Nedergaard J (2000). Norepinephrine induces vascular endothelial growth factor gene expression in brown adipocytes through a *β*-adrenoreceptor/cAMP/protein kinase A pathway involving Src but independently of Erk1/2. J. Biol. Chem.

[b16] Gao XM, Agrotis A, Autelitano DJ, Percy E, Woodcock EA, Jennings GL (2003). Sex hormones and cardiomyopathic phenotype induced by cardiac *β*_2_-adrenergic receptor overexpression. Endocrinology.

[b17] Giebler HA, Lemasson I, Nyborg JK (2000). p53 recruitment of CREB binding protein mediated through phosphorylated CREB: a novel pathway of tumor suppressor regulation. Mol. Cell. Biol.

[b18] Giordano FJ, Gerber HP, Williams SP, VanBruggen N, Bunting S, Ruiz-Lozano P (2001). A cardiac myocyte vascular endothelial growth factor paracrine pathway is required to maintain cardiac function. Proc. Natl Acad. Sci. USA.

[b19] Green DR, Kroemer G (2009). Cytoplasmic functions of the tumour suppressor p53. Nature.

[b20] Harikrishnan KN, Chow MZ, Baker EK, Pal S, Bassal S, Brasacchio D (2005). Brahma links the SWI/SNF chromatin-remodeling complex with MeCP2-dependent transcriptional silencing. Nat. Genet.

[b21] Heineke J, Auger-Messier M, Xu J, Oka T, Sargent MA, York A (2007). Cardiomyocyte GATA4 functions as a stress-responsive regulator of angiogenesis in the murine heart. J. Clin. Invest.

[b22] Hilfiker-Kleiner D, Kaminski K, Podewski E, Bonda T, Schaefer A, Sliwa K (2007). A cathepsin D-cleaved 16 kDa form of prolactin mediates postpartum cardiomyopathy. Cell.

[b23] Iaccarino G, Cipolletta E, Fiorillo A, Annecchiarico M, Ciccarelli M, Cimini V (2002). *β*_2_-adrenergic receptor gene delivery to the endothelium corrects impaired adrenergic vasorelaxation in hypertension. Circulation.

[b24] Iaccarino G, Ciccarelli M, Sorriento D, Galasso G, Campanile A, Santulli G (2005). Ischemic neoangiogenesis enhanced by *β*_2_-adrenergic receptor overexpression: a novel role for the endothelial adrenergic system. Circ. Res.

[b25] Jones JM, Petrofski JA, Wilson KH, Steenbergen C, Koch WJ, Milano CA (2004). beta2 adrenoceptor gene therapy ameliorates left ventricular dysfunction following cardiac surgery. Eur. J. Cardiothorac. Surg.

[b26] Kasper LH, Lerach S, Wang J, Wu S, Jeevan T, Brindle PK (2010). CBP/p300 double null cells reveal effect of coactivator level and diversity on CREB transactivation. EMBO J.

[b27] Kiriakidis S, Andreakos E, Monaco C, Foxwell B, Feldmann M, Paleolog E (2003). VEGF expression in human macrophages is NF-*κ*B-dependent: studies using adenoviruses expressing the endogenous NF-*κ*B inhibitor I*κ*B*α* and a kinase-defective form of the I*κ*B kinase 2. J. Cell Sci.

[b28] Lee JS, Jang DJ, Lee N, Ko HG, Kim H, Kim YS (2009). Induction of neuronal vascular endothelial growth factor expression by cAMP in the dentate gyrus of the hippocampus is required for antidepressant-like behaviors. J. Neurosci.

[b29] Ling H, Zhang T, Pereira L, Means CK, Cheng H, Gu Y (2009). Requirement for Ca^2+^/calmodulin-dependent kinase II in the transition from pressure overload-induced cardiac hypertrophy to heart failure in mice. J. Clin. Invest.

[b30] Liu L, Simon MC (2004). Regulation of transcription and translation by hypoxia. Cancer Biol. Ther.

[b31] Martini D, Monte MD, Ristori C, Cupisti E, Mei S, Fiorini P (2011). Antiangiogenic effects of *β*_2_-adrenergic receptor blockade in a mouse model of oxygen-induced retinopathy. J. Neurochem.

[b32] May D, Gilon D, Djonov V, Itin A, Lazarus A, Gordon O (2008). Transgenic system for conditional induction and rescue of chronic myocardial hibernation provides insights into genomic programs of hibernation. Proc. Natl Acad. Sci. USA.

[b33] Milano CA, Allen LF, Rockman HA, Dolber PC, McMinn TR, Chien KR (1994). Enhanced myocardial function in transgenic mice overexpressing the *β*_2_-adrenergic receptor. Science.

[b34] Pasquier E, Street J, Pouchy C, Carre M, Gifford AJ, Murray J (2013). *β*-blockers increase response to chemotherapy via direct antitumour and anti-angiogenic mechanisms in neuroblastoma. Br. J. Cancer.

[b35] Patterson AJ, Zhu W, Chow A, Agrawal R, Kosek J, Xiao RP (2004). Protecting the myocardium: a role for the *β*_2_ adrenergic receptor in the heart. Crit. Care Med.

[b36] Perez-Sayans M, Somoza-Martin JM, Barros-Angueira F, Diz PG, Gandara Rey JM, Garcia-Garcia A (2010). *β*-adrenergic receptors in cancer: therapeutic implications. Oncol. Res.

[b37] Rengo G, Zincarelli C, Femminella GD, Liccardo D, Pagano G, de Lucia C (2012). Myocardial *β*_2_-adrenoceptor gene delivery promotes coordinated cardiac adaptive remodelling and angiogenesis in heart failure. Br. J. Pharmacol.

[b38] Sano M, Minamino T, Toko H, Miyauchi H, Orimo M, Qin Y (2007). p53-induced inhibition of Hif-1 causes cardiac dysfunction during pressure overload. Nature.

[b39] Sheridan DJ, Autelitano DJ, Wang B, Percy E, Woodcock EA, Du XJ (2000). *β*_2_-adrenergic receptor overexpression driven by alpha-MHC promoter is downregulated in hypertrophied and failing myocardium. Cardiovasc. Res.

[b40] Swaminathan PD, Purohit A, Hund TJ, Anderson ME (2012). Calmodulin-dependent protein kinase II: linking heart failure and arrhythmias. Circ. Res.

[b41] Taimeh Z, Loughran J, Birks EJ, Bolli R (2013). Vascular endothelial growth factor in heart failure. Nat. Rev. Cardiol.

[b42] Talan MI, Ahmet I, Xiao RP, Lakatta EG (2011). *β*_2_AR agonists in treatment of chronic heart failure: long path to translation. J. Mol. Cell. Cardiol.

[b43] Thaker PH, Han LY, Kamat AA, Arevalo JM, Takahashi R, Lu C (2006). Chronic stress promotes tumor growth and angiogenesis in a mouse model of ovarian carcinoma. Nat. Med.

[b44] Toko H, Takahashi H, Kayama Y, Oka T, Minamino T, Okada S (2010). Ca^2+^/calmodulin-dependent kinase IIdelta causes heart failure by accumulation of p53 in dilated cardiomyopathy. Circulation.

[b45] Tomiyasu K, Oda Y, Nomura M, Satoh E, Fushiki S, Imanishi J (2000). Direct intra-cardiomuscular transfer of *β*_2_-adrenergic receptor gene augments cardiac output in cardiomyopathic hamsters. Gene Ther.

[b46] Wu D, Zhau HE, Huang WC, Iqbal S, Habib FK, Sartor O (2007). cAMP-responsive element-binding protein regulates vascular endothelial growth factor expression: implication in human prostate cancer bone metastasis. Oncogene.

[b47] Xu Q, Lekgabe ED, Gao XM, Ming Z, Tregear GW, Dart AM (2008). Endogenous relaxin does not affect chronic pressure overload-induced cardiac hypertrophy and fibrosis. Endocrinology.

[b48] Xu Q, Dalic A, Fang L, Kiriazis H, Ritchie RH, Sim K (2011). Myocardial oxidative stress contributes to transgenic *β*-adrenoceptor activation-induced cardiomyopathy and heart failure. Br. J. Pharmacol.

[b49] Yamaoka M, Yamamoto T, Ikeyama S, Sudo K, Fujita T (1993). Angiogenesis inhibitor TNP-470 (AGM-1470) potently inhibits the tumor growth of hormone-independent human breast and prostate carcinoma cell lines. Cancer Res.

[b50] Zhang D, Ma QY, Hu HT, Zhang M (2010). *β*_2_-adrenergic antagonists suppress pancreatic cancer cell invasion by inhibiting CREB, NFkappaB and AP-1. Cancer Biol. Ther.

[b51] Zhu W, Petrashevskaya N, Ren S, Zhao A, Chakir K, Gao E (2012). Gi-biased *β*_2_AR signaling links GRK2 upregulation to heart failure. Circ. Res.

